# Proteomic Profiling of *Burkholderia thailandensis* During Host Infection Using Bio-Orthogonal Noncanonical Amino Acid Tagging (BONCAT)

**DOI:** 10.3389/fcimb.2018.00370

**Published:** 2018-10-23

**Authors:** Magdalena Franco, Patrik M. D'haeseleer, Steven S. Branda, Megan J. Liou, Yasmeen Haider, Brent W. Segelke, Sahar H. El-Etr

**Affiliations:** ^1^Lawrence Livermore National Laboratory, Livermore, CA, United States; ^2^Sandia National Laboratories, Livermore, CA, United States

**Keywords:** *Burkholderia*, BONCAT, orthogonal amino acid labeling, intracellular pathogen, host infection, protein enrichment, proteome profiling, quorum sensing (QS)

## Abstract

*Burkholderia pseudomallei* and *B. mallei* are the causative agents of melioidosis and glanders, respectively, and are often fatal to humans and animals. Owing to the high fatality rate, potential for spread by aerosolization, and the lack of efficacious therapeutics, *B. pseudomallei* and *B. mallei* are considered biothreat agents of concern. In this study, we investigate the proteome of *Burkholderia thailandensis*, a closely related surrogate for the two more virulent *Burkholderia* species, during infection of host cells, and compare to that of *B. thailandensis* in culture. Studying the proteome of *Burkholderia* spp. during infection is expected to reveal molecular mechanisms of intracellular survival and host immune evasion; but proteomic profiling of *Burkholderia* during host infection is challenging. Proteomic analyses of host-associated bacteria are typically hindered by the overwhelming host protein content recovered from infected cultures. To address this problem, we have applied bio-orthogonal noncanonical amino acid tagging (BONCAT) to *B. thailandensis*, enabling the enrichment of newly expressed bacterial proteins from virtually any growth condition, including host cell infection. In this study, we show that *B. thailandensis* proteins were selectively labeled and efficiently enriched from infected host cells using BONCAT. We also demonstrate that this method can be used to label bacteria *in situ* by fluorescent tagging. Finally, we present a global proteomic profile of *B. thailandensis* as it infects host cells and a list of proteins that are differentially regulated in infection conditions as compared to bacterial monoculture. Among the identified proteins are quorum sensing regulated genes as well as homologs to previously identified virulence factors. This method provides a powerful tool to study the molecular processes during *Burkholderia* infection, a much-needed addition to the *Burkholderia* molecular toolbox.

## Introduction

*Burkholderia pseudomallei* and *B. mallei* are closely related Gram-negative bacteria that cause highly lethal disease (melioidosis and glanders, respectively) in humans and animals. Antibiotic treatment of infected patients is often unsuccessful due to the intrinsic resistance of both pathogens to a wide variety of antibiotics (Kenny et al., 1999; Larsen and Johnson, 2009; Schweizer, 2012; Wiersinga et al., 2012; Rhodes and Schweizer, 2016; Titball et al., 2017). Furthermore, to date there are no FDA-approved vaccines against these pathogens. Due to these factors, as well as their potential for deliberate aerosolization for airway delivery (Howe et al., 1971; Titball et al., 2008, 2017), these pathogens pose a high risk for misuse as bioweapons, and therefore are considered Tier 1 Select Agents by the Federal Select Agent Program at the Centers for Disease Control and Prevention (CDC).

*B. pseudomallei* and *B. mallei* are facultative intracellular bacteria that can persist and replicate within host cells, enabling them to evade many host defense mechanisms. Numerous virulence factors required for invasion of and replication within host cells have been identified (Stevens et al., 2002, 2004; Ulrich and DeShazer, 2004; Warawa and Woods, 2005; Ribot and Ulrich, 2006; Muangsombut et al., 2008; Galyov et al., 2010). Intracellular survival and circumvention of the immune system are also important determinants for the establishment of chronic infection (Nandi and Tan, 2013). Although the intracellular lifestyle of *Burkholderia* spp. is critical for bacterial survival within the host and the ultimate outcome of infection, the molecular processes that occur in intracellular bacteria are underexplored due to technical challenges and regulations controlling genetic manipulation of Select Agents. Proteomic analyses of host-associated bacteria are often confounded by the overwhelming amount of host proteins in the samples. The amount of protein derived from bacteria is several orders of magnitude lower than that derived from the host cells, and the dynamic range of even the state-of-the-art LC-MS/MS technologies is limited such that only a handful of the most abundant bacterial proteins can be identified without selective enrichment (Milo, 2013; Fels et al., 2017). In addition, bacterial proteins that mediate invasion of the host cell and survival within it (e.g., secreted effectors) are generally produced in low abundance and have short half-lives compared to most constituents of the bacterial proteome (Haraga et al., 2008a; Galán, 2009). For these reasons, selective enrichment of bacterial proteins is critical for efficient, in-depth analysis of the proteome expressed by bacteria during infection.

Previous attempts to selectively enrich the *Burkholderia* proteome during infection have relied on the recovery of intact bacteria from the infected cells through physical disruption and/or detergent solubilization of the host cells followed by filtration, differential centrifugation, flow cytometry, or immunoprecipitation for isolation of the bacteria (Becker et al., 2006; Shi et al., 2006; Grammel et al., 2010; Fels et al., 2017). However, physical isolation of the bacteria from host cells has its drawbacks, such as the need for large numbers of infected cells as starting material to enable isolation of sufficient numbers of intact bacteria, and the risk of proteome drift due to perturbation of the bacteria during their isolation. The recent advancement of selective labeling methods that enable enrichment of bacterial proteins from coculture offers a new solution that overcomes these technical challenges (Ngo et al., 2009; Tanrikulu et al., 2009; Grammel et al., 2010; Wang et al., 2010).

**Figure 1:**
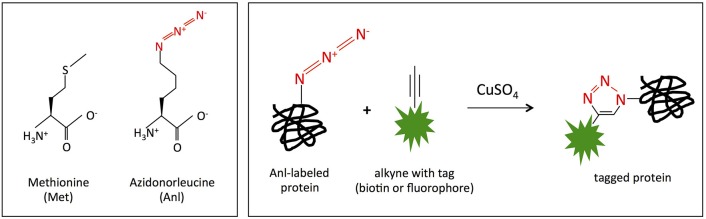
MetRS^NLL^ incorporates azidonorleucine (Anl) in place of methionine (Met). **(Left)** Structures of methionine (Met) and azidonorleucine (Anl). The engineered version of *E. coli* MetRS^NLL^ encodes three point mutations in the Met-binding pocket of methionyl-tRNA synthetase conferring high and preferential affinity for Anl resulting in loading of Anl onto Met-tRNA and into newly synthesized proteins in the place of Met. **(Right)** Copper catalyzed cycloaddition reaction of Anl-labeled protein with alkyne conjugated to biotin/fluorescent tag resulting in covalently tagged protein.

In order to identify the proteins expressed by *Burkholderia* spp. during host cell infection, we adapted bio-orthogonal noncanonical amino acid tagging (BONCAT), previously established for the intracellular pathogens *Yersinia enterocolitica, Salmonella typhimurium, Mycobacterium tuberculosis*, and *Toxoplasma gondii* (Grammel et al., 2010; Mahdavi et al., 2014; Chande et al., 2015; Wier et al., 2015) to use with *B. thailandensis*. BONCAT enables specific labeling and subsequent enrichment of newly expressed bacterial proteins in infected host cells. Protein labeling is achieved by expression of an engineered methionyl-tRNA synthetase (MetRS^NLL^) within the bacteria, which preferentially incorporates non-natural azidonorleucine (Anl) rather than methionine (Met) in newly translated proteins. Anl is loaded on tRNA by MetRS^NLL^ and not the wild-type MetRS, so only cells expressing MetRS^NLL^ incorporate azidonorleucine (Tanrikulu et al., 2009; Grammel et al., 2010; Mahdavi et al., 2014; Chande et al., 2015; Wier et al., 2015). The azide group on azidonorleucine can then be covalently modified with an affinity tag *via* click chemistry (copper catalyzed cycloaddition reaction), thereby enabling enrichment of tagged proteins from virtually any growth condition (Figure 1), including host intracellular compartments.

For the purpose of this study, we have generated a strain of *B. thailandensis* E264 that expresses the MetRS^NLL^ gene. *B. thailandensis* is closely related to *B. pseudomallei* and *B. mallei*, and while mildly pathogenic to immunocompetent humans, it is highly virulent *in vitro* and in animal models of infection (Brett et al., 1997, 1998; Haraga et al., 2008b; Galyov et al., 2010). In fact, *B. thailandensis* expresses homologs of many of the known virulence factors of the more pathogenic *Burkholderia* species, and is thought to employ the same molecular strategies to replicate inside host cells and to spread from cell to cell (Smith et al., 1997; Harley et al., 1998; Rainbow et al., 2002; Kespichayawattana et al., 2004; Stevens et al., 2005; DeShazer, 2007; Haraga et al., 2008b; Galyov et al., 2010). For these reasons, *B. thailandensis* is commonly used as a surrogate for *B. pseudomallei* and *B. mallei*. As an additional advantage, *B. thailandensis* requires only biosafety level 2 (BSL-2) containment and is exempt from the Select Agent regulations that limit distribution and genetic manipulation of its more virulent relatives.

Here we report that BONCAT can be used for selective labeling and enrichment of *Burkholderia* proteins from infected host cells for downstream proteomic analyses, as well as for *in situ* labeling of bacteria for visualization *via* fluorescence microscopy. Furthermore, we present a global proteomic profile of bacteria during host cell infection. This study reveals proteins that are differentially regulated in infection condition as compared to the bacterial monoculture condition. Among the identified proteins are those encoded by quorum sensing regulated genes, as well as homologs to previously identified virulence factors. The genetically modified strain of *B. thailandensis* (Bt-MetRS^NLL^) represents a powerful new tool for elucidating the biology and pathogenesis of *Burkholderia* spp.

## Materials and methods

### Expression of MetRS^NLL^ in *Burkholderia thailandensis*

*Burkholderia thailandensis* (Bt) strain E264 (ATCC 700388) (Brett et al., 1998) was transformed with a MetRS^NLL^ encoding plasmid in order to generate strain Bt-MetRS^NLL^. The MetRS^NLL^ gene sequence of *E. coli* (Mahdavi et al., 2014) was codon optimized for expression in *Burkholderia* spp. (Thermo Fisher), and placed under control of the P_*S*12_ promoter (Yu and Tsang, 2006) (Thermo Fisher). Additionally, the PstI restriction site within the coding region was replaced with a nucleotide sequence that preserved the codons and reading frame but destroyed the site, so that PstI could be used for cloning. The modified MetRS^NLL^ cassette was then cloned into a Mini-Tn7-kan vector (Norris et al., 2009), using the PstI and KpnI restriction sites. The correct MetRS^NLL^ sequence and proper integration into the Mini-Tn7-kan vector was confirmed by sequencing using the primers: 5′-CGCGTTGGCCGATTCATTAAT-3′, 5′-GCTACCAGCGCATGCG-3′, 5′-CGTTCGACGAGTACTGGAAG-3′, 5′-GCTCGATGGGCATCAACC-3′, 5′-GCGTCAGCATGATCG-3′ (Elim Biopharmaceuticals Inc.). *B. thailandensis* E264 was transformed with the Mini-Tn7-kan-MetRS^NLL^ plasmid *via* four-parental mating as previously described (Choi and Schweizer, 2006; Choi et al., 2006), using bacterial strains SM10(λ*pir*)/pTNS2; HB101/pRK2013; E1889 carrying the mini-Tn7-kan-MetRS^NLL^ delivery vector; and *B. thailandensis* E264. Colonies that grew on LB plates supplemented with 4 % glycerol, kanamycin (1,000 μg mL^−1^), and gentamicin (50 μg mL^−1^) were propagated on LB/kanamycin (1,000 μg mL^−1^) plates. Proper integration of kan-MetRS^NLL^ into the two attTn7 sites downstream of genes glmS1 and glmS2 was confirmed using the primer pairs glmS1DN/Tn7L (5′- GTTCGTCGTCCACTGGGATCA-3′/5′-ATTAGCTTACGACGCTACACCC-3′) and glmS2DN/Tn7L (5′-AGATCGGATGGAATTCGTGGAG-3′/5′-ATTAGCTTACGACGCTACACCC-3′).

### Host cell culture and infection

All infections were carried out using the A549 cell line (ATCC CCL-185), developed from a human lung epithelial carcinoma (Lieber et al., 1976), as the model host cell type. Host cells were cultured in Dulbecco's modified Eagle's medium (DMEM) with GlutaMAX^TM^ (Thermo Fisher) supplemented with 110 mg/L sodium pyruvate and 10 % fetal bovine serum. Host cell cultures were maintained at 37°C and 5% CO_2_ in a humidified incubator, and subcultured every 72 hrs. Nearly (90 %) confluent monolayers of host cells were infected at the specified multiplicity of infection (MOI), using bacteria grown to log phase in LB broth. Infected host cells were washed with phosphate-buffered saline (PBS) and harvested at the time points specified in the text, and either lysed for protein extraction or fixed for fluorescent labeling. For bacterial survival during host infection experiments, bacteria from infected host cells were enumerated by lysing the host cells through treatment with 0.5% saponin for 5 min and then plating dilutions of the lysate on LB agar.

### Incorporation of azidonorleucine (Anl) into Bt-MetRS^NLL^ proteins

Azidonorleucine (6-azido-L-lysine hydrochloride, Anl) (Baseclick, Germany) was incorporated into proteins expressed by Bt-MetRS^NLL^ bacteria grown in LB broth or in cocultures with host cells. Stock solution of 200 mM Anl was prepared in water and filter sterilized using 0.22 μm centrifuge tube filters (Corning). Labeling was performed by adding Anl to culture media at a final concentration of 1 mM Anl for 3–24 hrs.

### Protein extraction and cycloaddition reaction

Bacteria grown in LB broth were harvested by 10 min centrifugation at 3200 rcf at 4°C. To harvest proteins from infected host cells, monolayers of host cells in T175 flasks were washed 5x with 10 mL PBS and then collected using a cell scraper into 10 mL cold PBS, followed by 5 min centrifugation at 1500 rcf at 4°C. Cell pellets were flash frozen using dry ice, and freeze-thawed twice *via* room temperature/dry ice cycles. Pellets were then resuspended using 2% sodium dodecyl sulfate (SDS), 150 mM NaCl, 50 mM Tris HCl pH 8 buffer supplemented with EDTA-free Halt protease inhibitor cocktail (Thermo Fisher) and vortexed at 60°C for 1 h. Lysates were subjected to sonication (ten 1 s pulses at 50% power using Heat Systems Ultrasonics sonicator, model W-385) and spun down at 15,000 rcf for 5 min. Supernatants were transferred to new tubes and subjected to click reaction using the Click-IT Protein Reaction Buffer Kit (Thermo Fisher), using biotin alkyne to tag Anl-labeled proteins.

### SDS-PAGE and western blot analysis

Biotin-tagged lysates were combined with Laemmli loading buffer (BioRad) and boiled at 95°C for 5 min. Samples were loaded onto Mini-protean TGX precast gels 4–15% (BioRad) and subjected to 120 V for 1 h. Gels were either stained with SYPRO Ruby Protein Gel Stain (Thermo Fisher) or transferred to a nitrocellulose membrane (BioRad). Membranes were blocked in Tris-buffered saline with 0.05% Tween 20 (TBS-T) and 5% nonfat dry milk for 1 hr or overnight. Blots were hybridized with 1:10,000 Pierce™ High Sensitivity Streptavidin-HRP (Thermo Fisher) in 5% milk TBS-T buffer for 1 h. After three 5 min TBS-T washes, membranes were developed using SuperSignal^TM^ West Pico PLUS Chemiluminescent Substrate (Thermo Fisher). To stain for glyceraldehyde 3-phosphate dehydrogenase (GAPDH), blots were stripped using Restore^TM^ Western Blot Stripping Buffer for 10 min and blocked in 5% milk TBS-T buffer for 1 h. 1:500 dilution of primary rabbit anti-GAPDH antibody (Abcam) was hybridized with the blot for 1 h, followed by three 5 min TBS-T washes and then staining with 1:1,000 dilution of secondary goat anti-rabbit conjugated to HRP (Abcam). *Burkholderia* protein staining was accomplished using a cocktail of primary goat anti-*B. pseudomallei* (BEI DD-328) and goat anti-*B. mallei* (BEI DD-327) antibodies diluted to 1:500 in 5% milk TBS-T and 1:1,000 dilution of secondary donkey anti-goat antibodies conjugated to HRP (Abcam).

### Fluorescent labeling of host-associated bacteria using click chemistry

Nearly (90%) confluent monolayers of A549 cells grown on glass coverslips were infected with an overnight culture of Bt-MetRS^NLL^ at an MOI of 100. Simultaneously, 1 mM Anl was added to the infected cultures. After 6 h of growth, infected monolayers were washed five times with PBS pH 7.4 to remove unassociated extracellular bacteria and residual media, followed by fixation using 3.2% formaldehyde-PBS for 15 min at room temperature. Samples were washed once with PBS, and then blocked using 3% BSA-PBS overnight at 4°C. Host cells were visualized by labeling with 10 μg/mL Alexa Fluor 594-wheat germ agglutinin (WGA) conjugate (Thermo Fisher) in 3% BSA-PBS for 30 min. The coverslips were washed five times with PBS, and the monolayers permeabilized using 0.2% Triton X-100-3% BSA-PBS for 10 min at room temperature followed by one wash with 3% BSA-PBS. Click reaction was performed by incubating cells in Click-iT Cell Reaction Buffer (Thermo Fisher) with 2.5 μM Alexa Fluor 488-alkyne (Thermo Fisher) for 1 h in the dark at room temperature. The coverslips were washed three times with 3% BSA-PBS to remove any unreacted alkyne, and mounted on glass slides using Vectashield with DAPI (4′,6-diamidino-2-phenylindole) stain (Vector Laboratories) to detect host cell nuclei. Fluorescence was observed using a fluorescence microscope (Leica, DM5000B), and captured images were analyzed using ImageJ software.

### Streptavidin purification of Anl-labeled proteins

Following the click reaction, proteins were precipitated with acetone and washed twice with ethanol. The pellet was solubilized by vortexing samples at 60°C for 20 min in 4% SDS buffer supplemented with EDTA-free Halt protease inhibitor cocktail (Thermo Fisher). Samples were diluted with PBS supplemented with protease inhibitors to bring the concentration of SDS down to 1%, and filtered using 10 K MWCO concentrators (Pierce) at 10,000 rcf for 15 min at 40°C (increased temperature was necessary to prevent fouling of filters with precipitated SDS and protein). Samples were diluted up to 500 μL with PBS with 0.1% Tween 20; 100 μL of each input sample was stored at −20°C, while the rest of the sample was subjected to affinity purification using MyOne^TM^ Streptavidin C1 Dynabeads (Thermo Fisher) following the manufacturer's protocol. The entire unbound fraction was stored at −20°C; the beads were washed three times with 1 mL PBS +0.1% Tween 20, and bound protein eluted by boiling the beads for 10 min in 40–80 μL 1.5x NuPAGE^TM^ LDS Sample buffer (Thermo Fisher). To achieve high protein yield in the eluates from infected host cell samples, beads were aliquoted and proteins eluted by boiling in the same 20 μL of elution buffer. Input, unbound, and eluate fractions were subjected to SDS-PAGE and Western blot analysis as described above.

### Sample preparation for MS

Sample and data processing was performed by MS Bioworks (Ann Arbor, Michigan) as follows. 20 μg of each sample was processed by SDS-PAGE using a 10% Bis-Tris NuPAGE gel (Invitrogen) with the MES buffer system and run approximately 2 cm. The gel lane was excised robotically into 10 equal sized segments, and in-gel digestion was performed on each segment (ProGest, DigiLab) with the following protocol: A 50 μL 5 min 25 mM ammonium bicarbonate wash followed by 50 μL 5 min acetonitrile wash; 30 min reduction with 10 mM dithiothreitol at 60°C followed by 45 min alkylation with 50 mM iodoacetamide at room temperature; digestion with 25 ng trypsin (Promega) at 37°C for 4 hrs, and quenching with 0.1% formic acid. Samples were then lyophilized and reconstituted in 0.1% trifluoroacetic acid.

### Mass spectrometry

Fifty percent of each gel digest was analyzed by nano LC-MS/MS with a Waters NanoAcquity HPLC system interfaced to a ThermoFisher Q Exactive hybrid quadrupole-Orbitrap. 1 μg of peptides were loaded on a trapping column and eluted over a 75 μm analytical column at 350 nL/min with a binary gradient; solvent A was 0.1% formic acid, solvent B was 0.1% formic acid in acetonitrile. The gradient was 0 min 98% A, 13 min 82% A, 18 min 65% A, 19 min 50% A, 19.5 min 15% A, 20 min 98% A. The trap and analytical columns were packed with Luna C18 resin (Phenomenex). The mass spectrometer was operated in data-dependent mode, with the Orbitrap operating at 60,000 FWHM and 17,500 FWHM for MS and MS/MS respectively. The 15 most abundant ions were selected for MS/MS.

### MS data processing

Data were analyzed using Mascot with the following parameters: Enzyme: Trypsin/P; Database: SwissProt Human and UniProt *Burkholderia thailandensis* (strain ATCC 700388 / DSM 13276 / CIP 106301 / E264) (Proteome ID UP000001930, 5561 proteins) (forward and reverse appended with common contaminants); Fixed modification: Carbamidomethyl (C); Variable modifications: Oxidation (M), Acetyl (N-term), Pyro-Glu (N-term Q), Deamidation (N/Q), Met->Anl(M), Met->Anl-Biotin (M); Mass values: Monoisotopic; Peptide Mass Tolerance: 10 ppm; Fragment Mass Tolerance: 0.02 Da; Max Missed Cleavages: 2; Mascot DAT files were parsed into Scaffold Proteome Software for validation, filtering and to create a non-redundant list per sample. Data were filtered using 1% protein and peptide FDR and requiring at least two unique peptides per protein. The mass spectrometry proteomics data have been deposited to the ProteomeXchange Consortium *via* the PRIDE (Vizcaíno et al., 2016) partner repository with the dataset identifier PXD011042 and 10.6019/PXD011042.

### Differential protein expression analysis

Spectral count data from Mascot was analyzed using DESeq2 (Love et al., 2014) to identify proteins with at least a 2-fold differential expression between *B. thailandensis* in monoculture *vs*. host infection, and a FDR-adjusted *p*-value of 0.05 or less. Predicted operon structure was retrieved from DOOR - the Database for prOkaryotic OpeRons (Dam et al., 2007; Mao et al., 2009). Gene associations, and overrepresentation of pathways and GO functional categories within these significantly differentially expressed proteins were analyzed by mapping the *B. thailandensis* proteins to the homologous *B. pseudomallei* proteins in STRING (Szklarczyk et al., 2017). To review function annotations of select proteins, sequences were BLASTed against the Protein Data Bank (PDB; http://www.rcsb.org/pdb/) (Berman et al., 2000) to retrieve structural information, and against the core dataset of the Virulence Factors Database (VFDB; http://www.mgc.ac.cn/VFs/) (Chen et al., 2016) to identify homologs of known virulence factors.

## Results

### *B. thailandensis* strain Bt-MetRS^NLL^ incorporates anl into newly synthesized proteins

**Figure 2:**
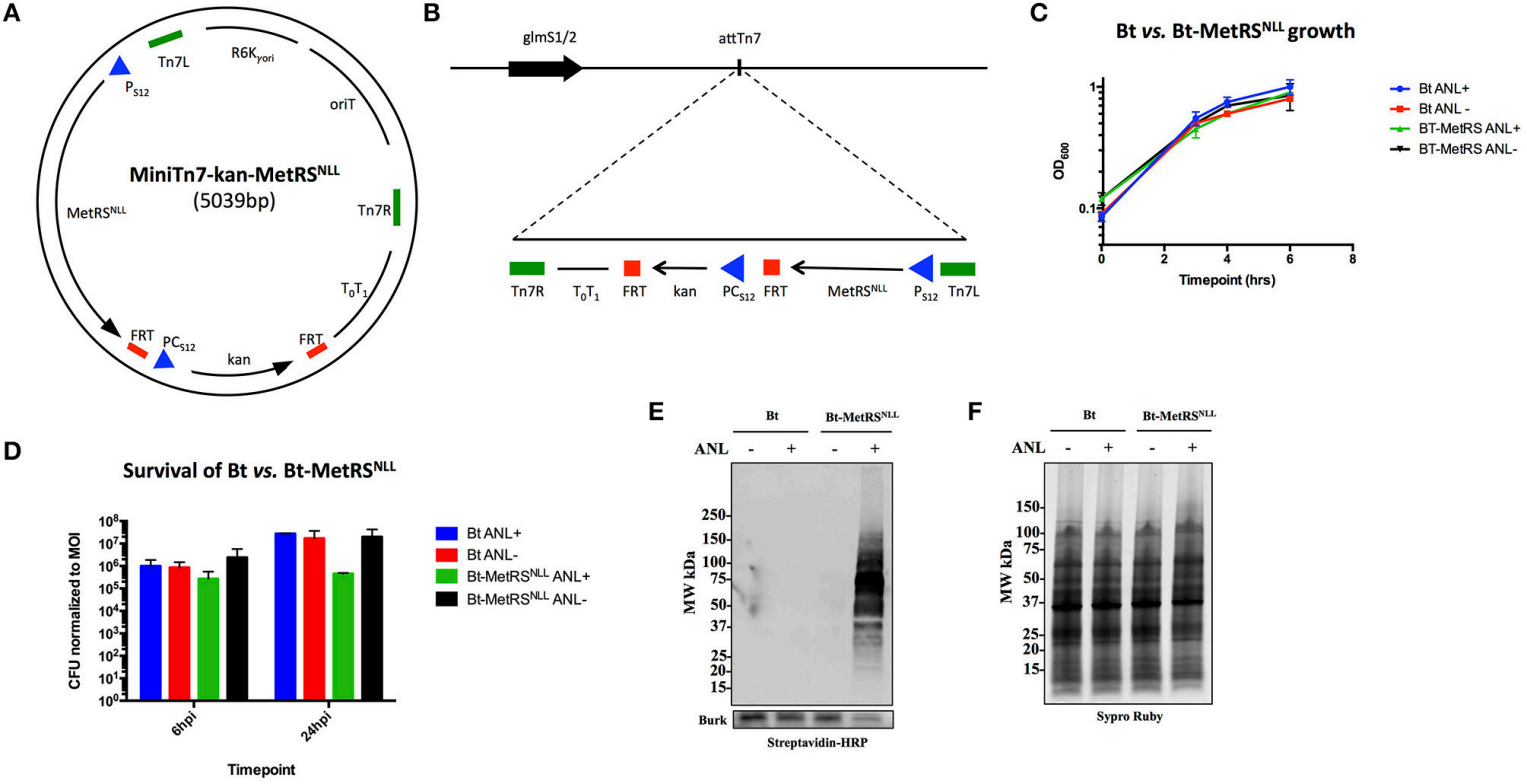
Expression of MetRS^NLL^ in *B. thailandensis* (Bt) leads to incorporation of Anl into Bt proteins. **(A)** Map of MiniTn7-kan-MetRS^NLL^ plasmid used for integration of MetRS^NLL^ into the genome of *B. thailandensis* strain E264. *E. coli* MetRS^NLL^ gene, optimized for expression in *Burkholderia* spp., is constitutively expressed using the P_S12_ promoter. PC_S12_ promoter drives the constitutive expression of kanamycin resistance used for selection of transformed bacteria. **(B)** Scheme of Tn7 transposon attachment sites downstream of glucosamine-6-phosphate synthetase genes 1 and 2 (glmS1/2), each located on one of the two *B. thailandensis* chromosomes, allowing for site-specific directional transposition of genes into *B. thailandensis* genome. FRT, flippase recognition target sites for flippase-mediated excision of FRT flanked DNA; P_S12_, *B. thailandensis* ribosomal protein S12 gene promoter; PC_s12_, *B. cenocepacia rpsL* promoter; T_0_T_1_, transcriptional terminator; Tn7L and Tn7R, left and right transposase recognition sites of Tn7 transposon; R6K_⋎*ori*_, origin of replication; oriT, conjugal origin of transfer. Black arrows indicate genes and their transcriptional orientations (MetRS^NLL^, methionyl-tRNA synthetase; kan, kanamycin resistance; glmS, glucosamine-6 phosphate synthetase). **(C)** Growth curves of wild type Bt and Bt-MetRS^NLL^ cultured in LB broth with or without azidonorleucine (Anl). Optical density (OD_600_) measurements of cultures were taken during log phase. Each growth curve represents two biological replicates. **(D)** Survival of bacteria (wild-type Bt and Bt-MetRS^NLL^ strains) assessed by infecting human epithelial cells (A549) at MOI 10. Cultures were grown in DMEM supplemented with or without Anl. At 2 h hpi, cells were washed with PBS to remove unassociated extracellular bacteria. Cell layers were washed and lysed with saponin at 6 and 24 hpi and bacterial counts determined by plating culture dilutions on LB agar. Colony forming units (CFUs) were normalized to the respective MOI for each strain. **(E)** Incorporation of Anl in Bt-MetRS^NLL^ proteins was confirmed by subjecting lysates of Bt or Bt-MetRS^NLL^ cultured in broth with or without Anl to click reaction using biotin-alkyne. Biotinylated proteins were detected by Western blot stained using streptavidin-HRP. Primary goat anti-*Burkholderia* antibodies and secondary donkey anti-goat antibodies conjugated to HRP were used to stain the blot as a loading control. **(F)** Relative protein quantity in lysates from E was assessed by loading the same amounts of lysates onto SDS-PAGE gel and detecting total protein using Sypro Ruby stain.

To test whether the orthogonal amino acid labeling method can be used to label newly synthesized proteins in *Burkholderia* spp., *E. coli* methionyl-tRNA synthetase MetRS^NLL^ was expressed in *B. thailandensis* strain (Bt) E264. Since *Burkholderia* genomes have higher GC nucleotide content compared to that of *E. coli*, the MetRS^NLL^ gene from *E. coli* was optimized for expression in *Burkholderia* spp. by increasing the proportion of G and C nucleotides from 52 to 63% of total nucleotides in the DNA sequence without altering the translated amino acid sequence. The *B. thailandensis* promoter for the ribosomal protein S12 gene (P_s12_), which has been shown to drive constitutive expression of genes in *Burkholderia* spp. (Choi et al., 2008), was inserted upstream of the modified MetRS^NLL^ sequence in order to drive constitutive expression of MetRS^NLL^. The P_s12_ driven MetRS^NLL^ gene construct was cloned into the MiniTn7-kan plasmid backbone (Norris et al., 2009) resulting in MiniTn7-kan-MetRS^NLL^ plasmid (Figure 2A). The Mini-Tn7 transposon system developed by Choi et al. (2006) and Choi and Schweizer (2006) was used for its ability to introduce the MetRS^NLL^ cassette into the *B. thailandensis* genome in a site-specific and directional manner. Insertion of Tn7 occurs at attTn7 sites that are located downstream of essential and highly conserved glucosamine-6-phosphate synthetase gene (glmS) (Craig, 1991; Peters Craig and Craig, 2001a,b). The *B. thailandensis* genome contains one attTn7 site on each of its two chromosomes, downstream of the glmS1 and glmS2 genes (Choi et al., 2005). MiniTn7-kan-MetRS^NLL^ vector was delivered to *B. thailandensis* E264 *via* four-parental mating (Figure 2B). The resulting Bt-MetRS^NLL^ strain, which was used for the rest of our study, contains a single insertion at attTn7 site downstream of the glmS1 gene. However, it should be noted that our selection generated additional strains with MetRS^NLL^ integration at the glmS2 attTn7 site. Consistent with previous reports, we haven't observed a strain with integration at both transposon sites (Choi et al., 2006).

To assess the effects of MetRS^NLL^ expression on bacterial replication, Bt-MetRS^NLL^ and its parental strain were grown in LB broth in the presence or absence of Anl, and the optical density (OD_600_) of the cultures was measured during the logarithmic phase of growth (Figure 2C). Our results indicate that bacterial replication of Bt in broth culture is not affected by expression of the MetRS^NLL^ cassette, or by the presence of Anl in the culture media.

Since *Burkholderia* spp. are facultative intracellular bacteria and the goal of our study was to isolate proteins from *Burkholderia* infecting host cells, we next tested whether Bt-MetRS^NLL^ exhibits any deviations from the wild-type Bt strain with respect to survival. A previously published study that utilized the same labeling method in the human pathogen *Toxoplasma gondii* had reported a growth defect in Anl-labeled parasites during later timepoints of parasite infection of host cells (Wier et al., 2015). To test for Bt-MetRS^NLL^ growth defects, the human lung epithelial cell line A549 was infected with Bt-MetRS^NLL^ at a multiplicity of infection (MOI) of 10 and cultured in media supplemented with 1 mM Anl. At 2 h post infection (hpi), infected cells were washed thoroughly with PBS to remove extracellular bacteria. At 6 and 24 hpi, cells were washed and lysed with saponin and bacterial loads enumerated by plating dilutions on LB agar. The results showed that survival of Bt-MetRS^NLL^ is not significantly affected at 6 hpi compared to Bt and unlabeled controls (Figure 2D). At 24 hpi, however, the Bt-MetRS^NLL^ strain exhibited decreased survival relative to Bt. This decrease was not observed when Bt-MetRS^NLL^ was grown in media without Anl (Figure 2D). Consistent with the *Toxoplasma* study (Wier et al., 2015), these results suggest that Anl incorporation does not affect the growth of Bt during earlier timepoints; however, the fitness is decreased when labeled bacteria are grown for extended time periods inside the host cell. These results suggest that earlier timepoints could offer more relevant proteome snapshots for the purposes of proteomic profiling studies. Therefore, in the current study our proteomic analyses include samples derived from bacteria grown in host cells for no longer than 18 h.

To confirm that Bt-MetRS^NLL^ incorporates Anl into newly synthesized proteins, bacteria were grown overnight in 5 mL LB broth supplemented with 1 mM Anl and lysates of bacterial cultures were subjected to a cycloaddition reaction using biotin-conjugated alkyne. The presence of biotinylated proteins in lysates was detected by Western blotting using streptavidin conjugated to horseradish peroxidase (HRP), and HRP signal was compared across lysates obtained from wild-type Bt or Bt-MetRS^NLL^ strains grown with or without Anl. Our results confirmed the presence of biotin-tagged proteins in Bt-MetRS^NLL^ grown in Anl-supplemented media; and not in wild-type Bt, or in Bt-MetRS^NLL^ grown without Anl (Figures 2E,F). These results indicate that only the bacteria expressing MetRS^NLL^ can incorporate Anl into newly synthesized proteins. Similarly, the labeling efficiency was tested in lysates derived from bacteria grown in increasing concentrations of Anl (0-4 mM). Western blots of lysates obtained from these conditions showed no observable differences in labeling efficiency resulting from increased concentration of label (>1 mM) or by growing bacteria in culture media depleted of Met (data not shown).

### Anl-labeling of *Burkholderia* Bt-MetRS^NLL^ infecting host cells is bacteria-specific and allows for *in situ* fluorescent detection of host-associated bacteria

**Figure 3:**
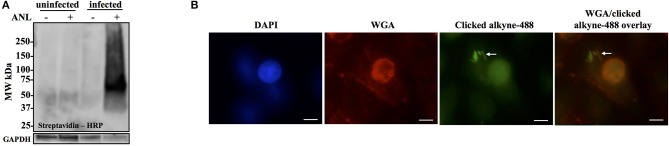
Anl-labeling of Bt-MetRS^NLL^ during infection is bacteria-specific and allows for *in-situ* fluorescent detection of host-associated bacteria. **(A)** Human epithelial cells (A549) were infected at MOI 100 and cultured for 18 hrs in DMEM media supplemented with or without 1 mM Anl. Lysates from infected and uninfected monolayers were subjected to click chemistry using alkyne conjugated to biotin. Biotin-tagged proteins in cell lysates were detected by Western blotting with streptavidin-HRP. As a loading control, human GAPDH was detected using primary rabbit anti-GAPDH antibodies and secondary goat anti-rabbit antibodies conjugated to HRP. **(B)** A549 cells were infected at an MOI of 100 with Bt-MetRS^NLL^ bacteria and grown in media supplemented with 1 mM Anl for 6 hrs. Infected cells were fixed and stained with Alexa Fluor 594-wheat germ agglutinin (WGA) conjugate to visualize host cell membranes (red). Cells were subjected to click chemistry using Alexa Fluor 488 conjugated to alkyne to tag Anl-labeled proteins (green). Host cell nuclei were stained using 6-diamidino-2-phenylindole (DAPI) (blue). White arrow indicates bacteria. Fluorescent signal was visualized using fluorescence microscopy; 100x magnification was used for all images. Scale bars indicate the distance of 10 μm.

To confirm that labeling occurs in bacteria growing within host cells, A549 cells were infected with Bt-MetRS^NLL^ at MOI 100 and in the presence of 1 mM Anl. At 1 h post infection (hpi), the cells were washed 5 times with 10 mL PBS and then incubated in fresh medium supplemented with Anl. At 18 hpi, infected monolayers were washed thoroughly with PBS to remove extracellular bacteria, harvested, and lysates subjected to cycloaddition reaction using biotin-conjugated alkyne. Western blot analysis of the samples revealed the presence of biotinylated proteins only in the cells infected with Bt-MetRS^NLL^ grown in the presence of Anl, while low signal was observed in samples containing lysates of infected cells without Anl or uninfected cells (Figure 3A). These data indicate that Bt-MetRS^NLL^ incorporates Anl during host infection, and that Anl-labeling is specific to bacterial proteins expressed by Bt-MetRS^NLL^.

To test the MetRS^NLL^ system utility for *in situ* fluorescent labeling of *Burkholderia* spp. during infection, A549 were infected with Bt-MetRS^NLL^ at an MOI of 100 and cultured in the presence of Anl. After 6 h of incubation, the infected monolayers were washed thoroughly to remove extracellular bacteria and then fixed with formaldehyde. The cells were permeabilized and subjected to the cycloaddition reaction, using Alexa Fluor 488-conjugated alkyne to tag Anl-labeled bacterial proteins. Host cell membranes were labeled with Alexa Fluor 594-wheat germ agglutinin (WGA) conjugate prior to permeabilization, and 4′,6-diamidino-2-phenylindole (DAPI) stain was used to label the host cell nuclei. Immunofluorescence microscopy revealed bright staining of AF488-labeled bacteria (Figure 3B). As expected, bacteria grown without Anl were not labeled (data not shown). A low fluorescent background was observed in infected and uninfected host cells that were not exposed to Anl, indicating that the fluorescent alkyne tag incorporates nonspecifically into fixed cells at low levels. Increased fluorescence signal was observed in bacteria pre-treated with 1 mM Anl prior to infection; however, pre-treatment was not necessary to detect the bacteria in infected host cells. These results indicate that the MetRS^NLL^ system enables *in situ* fluorescent labeling of *Burkholderia* spp. during infection of host cells.

### Anl tagging enables the enrichment of *Burkholderia* proteome

**Figure 4:**
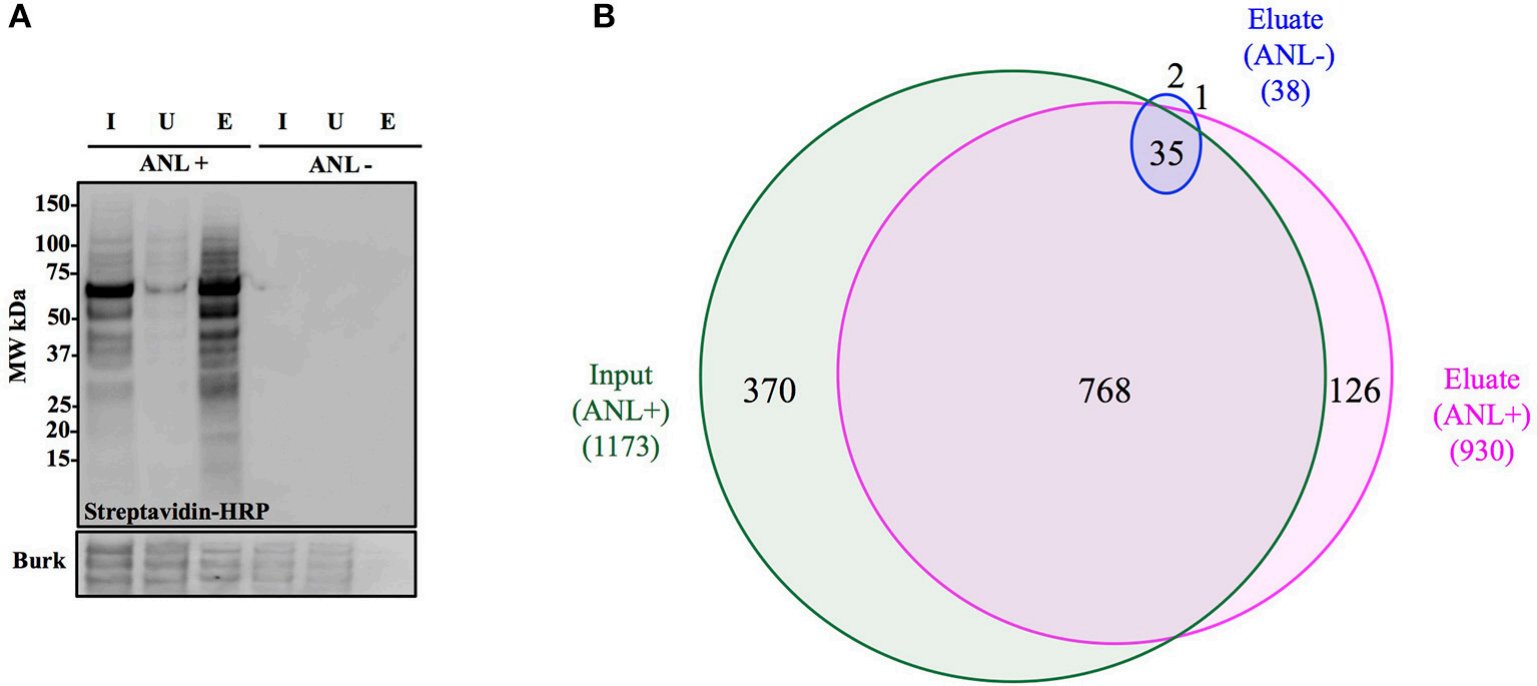
Anl-labeled proteins expressed by Bt-MetRS^NLL^ can be purified from bacterial lysates. **(A)** Bt-MetRS^NLL^ bacteria were grown for 4 hrs in DMEM media with or without Anl. Bacterial lysates were subjected to cycloaddition reaction using biotin-conjugated alkyne and biotin-tagged proteins were purified using magnetic streptavidin beads. Input (I), unbound (U), and eluate (E) samples obtained by affinity purification were analyzed by Western blotting using streptavidin-HRP to visualize biotin-tagged proteins. Primary goat anti-*Burkholderia* antibodies and secondary donkey anti-goat antibodies conjugated to HRP were used to determine relative bacterial protein abundance in samples. **(B)** Venn diagram representing the number of proteins identified by mass spectrometry in input and eluate fractions from Anl-labeled bacterial lysates and an eluate derived from unlabeled bacterial lysate.

To demonstrate capture of Anl-labeled proteins from bacterial lysates, Bt-MetRS^NLL^ bacteria were grown for 4 hrs in DMEM media containing 1 mM Anl or no Anl, lysates of the bacteria were subjected to the cycloaddition reaction using biotin-conjugated alkyne, and the biotin-tagged proteins were purified from the lysates using magnetic streptavidin beads. The analysis of input (total protein added to the beads), unbound (protein fraction that did not bind to the beads) and eluate (protein that bound to the beads) samples by Western blotting using streptavidin-HRP revealed that Anl-tagged proteins were present in the eluate fraction in amounts comparable to those of the input sample (Figure 4A). A small amount of biotin-tagged protein was observed in the unbound fraction. As expected, cell lysates from bacteria grown in media without Anl did not contain biotinylated proteins in any fraction. To compare the relative protein abundance in fractions from Anl+ and Anl- samples, the Western blot membranes were stained with primary goat anti-*Burkholderia* antibodies and secondary donkey anti-goat antibodies conjugated to HRP. The results indicate that the eluate fractions derived from the Anl-labeled Bt-MetRS^NLL^ samples contained large amounts of protein, as expected, but that the unbound fractions contained considerable amounts of protein as well. These data are consistent with the idea that Bt-MetRS^NLL^ does not incorporate Anl in all expressed proteins. As expected, the input and unbound fractions derived from the unlabeled Bt-MetRS^NLL^ contained similar amounts of protein, indicating that binding was specific to Anl-labeled proteins. In contrast, no protein was detected in the corresponding eluate fractions derived from the unlabeled Bt-MetRS^NLL^, indicating that there was little or no nonspecific binding of proteins to the beads. Taken together, these results indicate that Anl-labeled proteins can be efficiently and specifically recovered from bacterial lysates.

To determine the degree of proteome coverage that can be achieved with BONCAT, the input and eluate fractions obtained in the above experiment were analyzed by LC-MS/MS. A total of 1173 proteins were detected in the input fraction, and 930 proteins in the eluate fraction, with an overlap of 768 proteins, indicating a recovery efficiency of 68% (Figure 4B). Incomplete recovery of proteins detected in the input fraction was likely due to inefficient labeling of newly expressed proteins and/or the presence of proteins expressed before the addition of Anl. This finding agrees with the Western blot analysis results (Figure 4A), which indicate the presence of unlabeled protein in the unbound fraction. Of note is the observation that 126 proteins were detected in eluates but not the input samples of Anl-labeled bacterial lysates. These proteins were predominantly low abundance proteins, whose spectral counts may have been masked by more abundant proteins in the input sample.

To assess the degree of nonspecific recovery, we subjected unlabeled bacterial proteins (derived from Bt-MetRS^NLL^ grown in DMEM media without Anl) to affinity purification. LC-MS/MS analysis of input and eluate samples revealed that only 38 proteins, corresponding to 3.0% of the proteins detected in Anl-labeled lysates, were also detected in the eluates of unlabeled lysates (Figure 4B). This result is consistent with the Western blot result showing the absence of detectable protein in the eluate fraction of unlabeled Bt-MetRS^NLL^ lysate, indicating little nonspecific protein binding to the streptavidin column (Figure 4A). Taken together, these results indicate that orthogonal amino acid labeling of *Burkholderia* in culture enables interrogation of most of the pathogen's proteome, with little background contributed by nonspecific protein recovery.

### Selective enrichment of *Burkholderia* proteome from infected host cells

**Figure 5:**
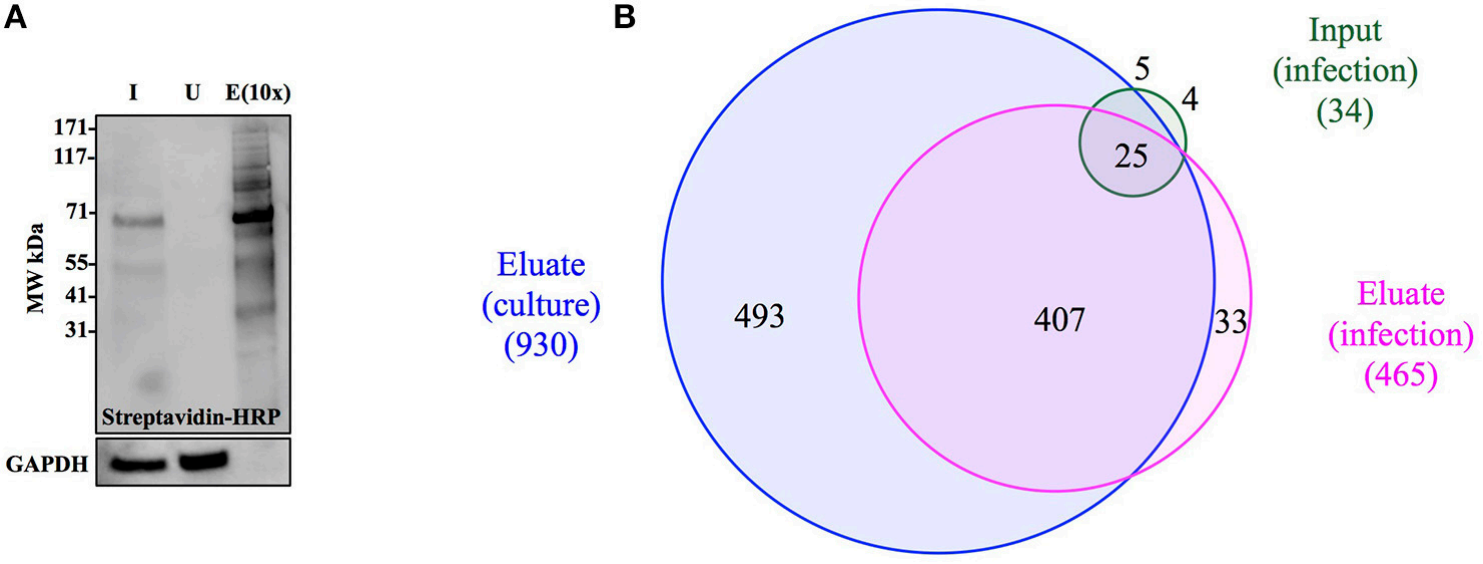
The enrichment of Anl-labeled Bt-MetRS^NLL^ from infected host cells *via* affinity purification. **(A)** A549 cells were infected with Bt-MetRS^NLL^ bacteria at MOI 50 and grown for 18 hrs in DMEM supplemented with Anl. Cell lysates were biotinylated *via* cycloaddition and subjected to affinity purification using streptavidin beads. Western blotting with streptavidin-HRP was used to detect tagged proteins in input (I), unbound (U), and eluate (E) fractions. The eluate fraction is concentrated 10x relative to the input. Primary rabbit anti-GAPDH antibodies and secondary goat anti-rabbit antibodies conjugated to HRP were used to assess the relative amount of host protein in samples. **(B)** Venn diagram representing bacterial proteins identified by mass spectrometry in input and eluate fractions that were derived from infected host cells. “Eluate (culture)” corresponds to bacterial proteins identified in lysates of bacteria grown in culture.

The main goal of this study was to develop a technique that allows for selective enrichment of proteins produced by *Burkholderia* during infection. To test whether this could be accomplished *via* BONCAT, A549 cells were infected with Bt-MetRS^NLL^ bacteria at an MOI of 50 and then grown for 18 hrs in media supplemented with Anl. The infected host cells were washed thoroughly to eliminate most extracellular bacteria, and cell lysates prepared using 2% SDS buffer. Cycloaddition reaction using biotin-alkyne probe followed by streptavidin purification was performed to enrich for biotin-tagged bacterial proteins. The eluate fraction was concentrated ~10x relative to the input, to enable detection of even low-abundance proteins. Input, unbound, and eluate fractions were subjected to SDS-PAGE and Western blotting analysis using streptavidin-HRP and chemiluminescence for protein detection. The results show that biotinylated proteins were readily detected in the input sample and eluate fraction, whereas no biotinylated proteins were detected in the unbound fraction (Figure 5A). These results indicate that Anl-labeled bacterial proteins were efficiently recovered from the infection culture. Moreover, GAPDH contributed by the host cells was detected only in the input and unbound fractions, at roughly similar levels, whereas none was detected in the eluate fraction, suggesting that affinity purification was highly specific to bacterial proteins.

To further assess the efficacy with which BONCAT enabled selective recovery of proteins produced by Bt-MetRS^NLL^ bacteria during infection, we used LC-MS/MS to analyze the input and eluate samples derived from the infection cultures. This resulted in identification of 465 *Burkholderia* proteins in the eluate fraction, as compared to 34 proteins in the input sample, an enrichment of ~14-fold (Figure 5B). In contrast, 1805 host proteins were identified in the input sample, whereas only 793 were identified in the eluate, a depletion of ~2-fold. Despite successful enrichment of the proteome produced by the bacteria during host infection, we consistently observed ~2-fold reduced proteome coverage as compared to that achieved by applying BONCAT to the bacteria in culture. This decrease in proteome coverage was possibly due to lower total protein concentrations in eluates recovered from infection cultures, as compared to those from bacterial monocultures. It is also possible that the host proteins from the infection cultures physically interfered with recovery of the bacterial proteins, perhaps during the biotin-tagging and/or affinity purification steps. In any case, coverage of the proteome produced by the bacteria infecting host was sufficient for robust comparative analysis with the proteome produced by bacteria in culture, as described below. Taken together, our results indicate that BONCAT can be employed to effectively enrich *Burkholderia* proteins from infected host cells, and that the recovered protein samples are compatible with proteomic analysis by mass spectrometry.

### Analysis of the proteome of *Burkholderia* during infection

**Figure 6:**
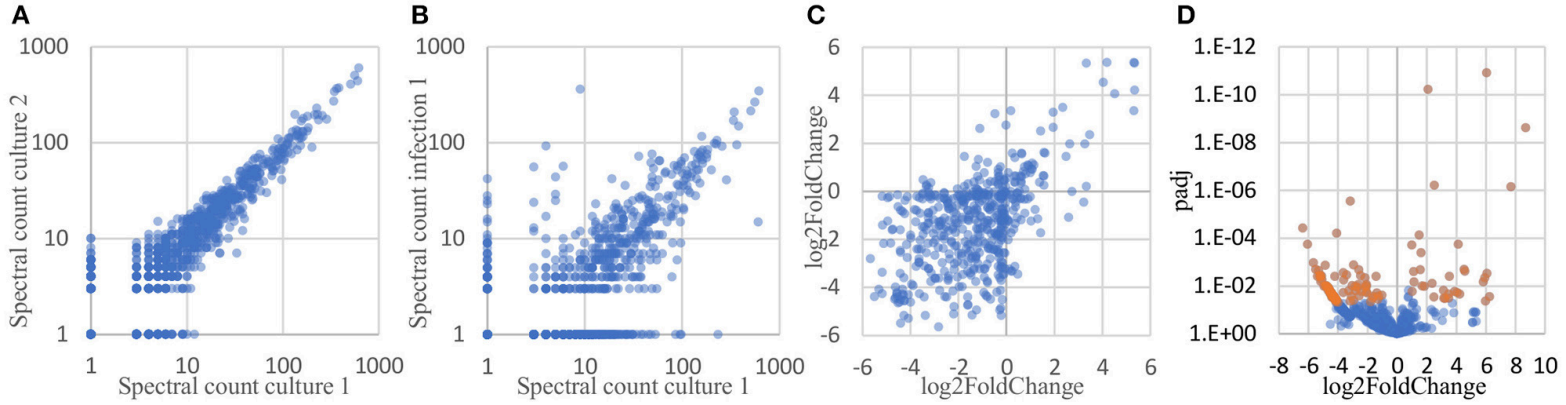
Spectral counts provide a highly reproducible relative quantitation of protein levels. **(A)** Spectral counts of biological replicates are highly correlated (average correlation = 0.97; average correlation between log-transformed spectral counts = 0.85). **(B)** Spectral counts of cultured *vs*. infection show substantial correlation as well (average = 0.77; average correlation between log-transformed spectral counts = 0.67). **(C)** Adjacent genes on the same operon show similar fold changes in protein levels, as calculated by DESeq2 (correlation = 0.53). **(D)** Volcano plot of the DESeq2 results, showing 125 proteins differentially expressed by at least 2-fold, with a FDR-adjusted *p*-value of 0.05 or better (shown in orange).

To determine which proteins are differentially expressed by *Burkholderia* during infection, proteomic datasets obtained from the two conditions of interest (in monoculture and host infection, as described above) were quantitatively analyzed and compared. Three biological replicates of each condition were included in this analysis. In total, 1171 proteins were identified by at least 2 peptide spectra in at least one of the samples, representing 21% of all coding sequences in the *B. thailandensis* genome (Supplementary Table 1). Spectral counting of peptides has been shown to be an effective method for relative quantification of proteins identified by MS/MS in label-free proteomics (Lundgren et al., 2010; Arike and Peil, 2014). We found that although the total number of spectra between some of the biological replicates differed by more than two-fold, correlation between the replicates is remarkably good, showing high reproducibility even across cultures generated in different weeks (Figure 6A). Likewise, even though the monoculture samples on average yielded two to three times as many total identified spectra as the host infection samples, we found a good correlation between replicates from the two different conditions (Figure 6B), suggesting that a majority of the detected proteins were expressed at similar levels in monoculture *vs*. in host cells.

Here, we used DESeq2 (Love et al., 2014) to normalize data sets from each samples to each other, calculate an overall log2 fold change between the two conditions for each protein, and generate an FDR-corrected *p*-value for the calculated differential expression. DESeq2 is applicable to proteomics data sets because spectral count data share some of the same statistical features and statistical analysis challenges as found in RNAseq read count data for transcriptomics: discrete counts, uneven numbers of counts *per* sample, highly skewed expression distributions, heteroscedastic noise, *etc*. Data sets with these features require robust normalization methods and sophisticated statistical analyses tools to correctly analyze differential protein expression. Conveniently, these parallels also mean that some of the mature analysis packages developed for RNAseq transcriptomics data can be applied directly to spectral count data as well (Langley and Mayr, 2015). Proteins encoded by adjacent genes that were predicted to belong to the same operon (Dam et al., 2007; Mao et al., 2009) were found to show similarities in differential expression, in both direction (up- *vs*. downregulation) and degree (fold change) (Figure 6C), providing independent evidence that spectral counts can be used to measure changes in protein expression. DESeq2 analysis identified 125 *Burkholderia* proteins that were differentially expressed by more than 2-fold with an FDR-corrected *p*-value of 0.05 or lower; including 33 proteins that were expressed at significantly higher levels in host-associated bacteria, and 92 proteins expressed at significantly lower levels in host-associated bacteria, as compared to the levels expressed in bacteria in monoculture (top 10 most over- and under-expressed in Table 1, Figure 6D, Supplementary Table 2).

**Table 1:** List of the top 10 upregulated and downregulated proteins under host infection condition relative to bacterial monoculture.

**Gene**	**Locus**	**Description**	**log2fold**	**padj**
Q2T3Y0	BTH_II1925	Chitin binding domain protein	8.700152	2.40E-09
Q2SY55	BTH_I1606	5-methyltetrahydropteroyltriglutamate–homocysteine methyltransferase	7.687991	7.22E-07
Q2T5L4	BTH_II1339	Uncharacterized protein	6.25127	0.028059
Q2T5P6	BTH_II1307	Uncharacterized protein	6.068985	0.003034
Q2T6D1	BTH_II1071	Uncharacterized protein	6.057949	1.23E-11
Q2T740	BTH_II0812	Serine carboxypeptidase family protein	5.971823	0.04308
Q2STH1	BTH_I3286	Membrane protein, putative	5.925939	0.004677
Q2SX64	BTH_I1956	Non-ribosomal peptide synthase domain protein	5.818015	0.006848
Q2T4X6	BTH_II1578	Microbial collagenase, putative	4.592752	0.002465
Q2T3X5	BTH_II1930	AMP-binding domain protein	4.524587	0.002044
Q2SU82	BTH_I3013	Glutamate synthase, NADH/NADPH, small subunit domain protein	−5.15795	0.006614
Q2T3Z7	BTH_II1908	Phosphoenolpyruvate phosphomutase	−5.17104	0.004039
Q2SVV3	BTH_I2425	Syringomycin biosynthesis enzyme, putative	−5.17506	0.003605
Q2SXA6	BTH_I1913	DNA mismatch repair protein MutS	−5.23481	0.004039
Q2T3C2	BTH_II2139	TonB-dependent heme/hemoglobin receptor family protein	−5.30296	0.002318
Q2STQ3	BTH_I3202	Acid phosphatase AcpA	−5.38116	0.004039
Q2SZP4	BTH_I1051	Sulfate transporter, putative	−5.50012	0.002044
Q2T0Y4	BTH_I0608	Cytochrome c family protein	−5.65984	0.001064
Q2SWD0	BTH_I2248	S1 RNA binding domain protein	−6.05624	0.000183
Q2SVV9	BTH_I2419	Cyclic peptide ABC transporter, ATP-binding protein	−6.37937	3.86E-05

Analysis of the protein interactions and biochemical pathways involving the 33 proteins overexpressed in host-associated bacteria revealed an association network consisting of enzymes involved in butanoate metabolism, and in valine, leucine and isoleucine degradation (Supplementary Figure 1A). Comparable analysis of the 92 proteins underexpressed in host-associated bacteria revealed a larger set of functional categories that encompassed over half of the proteins, including translation (14 proteins, including 11 ribosomal), biosynthesis of amino acids (11 proteins) and secondary metabolites (17 proteins), and iron uptake mechanisms (13 proteins) (Supplementary Figure 1B). Downregulation of translation and amino acid biosynthesis may indicate a slower growth rate of the Bt-MetRS^NLL^ strain at 18 hpi.

**Figure 7:**
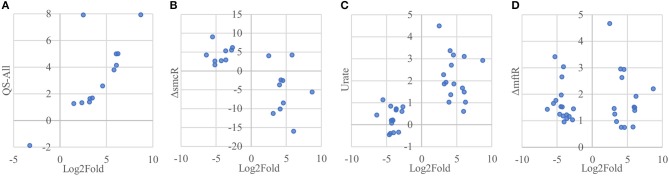
Protein overexpression in host-associated bacteria is correlated with gene expression of regulons involved in virulence. **(A)** Proteins encoded by genes upregulated by quorum sensing systems in *B. thailandensis* also tend to be overexpressed in host-associated bacteria. Here we show the genes differentially expressed when adding all three quorum sensing molecules to a mutant unable to produce them. **(B)** Proteins encoded by genes upregulated in an smcR deletion mutant also tend to be overexpressed in host-associated bacteria. **(C)** Proteins encoded by genes upregulated in the presence of urate also tend to be overexpressed in host-associated bacteria. **(D)** Genes upregulated in an mftR deletion mutant show no clear protein expression pattern in host-associated bacteria.

Of the 33 proteins overexpressed in host-associated bacteria, 17 (~52 %) are encoded by genes previously shown to be subject to quorum sensing control (Majerczyk et al., 2014), with the vast majority of them (15/17 genes) positively regulated by one or more of the three quorum sensing systems in *B. thailandensis* (Figure 7A, Supplementary Table 2). In contrast, only 5 of the 92 proteins underexpressed in host-associated bacteria (~5%) are encoded by genes controlled by quorum sensing; in all cases they are positively regulated by quorum sensing. The overexpression of quorum-regulated proteins during infection suggests the bacteria may be using quorum sensing to gauge how much they have replicated inside individual host cells.

Interestingly, the transcription factor ScmR (Q2SYQ0, encoded by BTH_I1403), a master regulator of genes mediating biosynthesis of secondary metabolites (Mao et al., 2017), was among the 15 proteins overexpressed in host-associated bacteria and encoded by genes positively regulated by quorum sensing. Moreover, we found that of the other 124 differentially expressed proteins, 19 (~15%) are encoded by genes that are regulated by ScmR (Figure 7B, Supplementary Table 2). As might be expected, all of the proteins encoded by ScmR-induced genes are overexpressed in host-associated bacteria (8 proteins), and most of those encoded by ScmR-repressed genes are underexpressed in host-associated bacteria (9/11 proteins). A notable exception to this trend is BtaC, which is encoded by a gene (BTH_II1224) belonging to the gene cluster that directs biosynthesis of bactobolins, a family of antibiotics (Seyedsayamdost et al., 2010). ScmR has been shown to negatively regulate expression of bactobolin biosynthesis genes (Mao et al., 2017), yet we found that like ScmR itself, BtaC is overexpressed in host-associated bacteria, indicating that in the infection context, BtaC levels are not determined by ScmR-mediated transcriptional repression alone.

We found that 16 of the 33 proteins overexpressed in host-associated bacteria are encoded by genes that are upregulated in the presence of 5 mM urate (Gupta et al., 2017; Figure 7C, Supplementary Table 2). In contrast, only one of the 92 proteins underexpressed in host-associated bacteria is encoded by a gene upregulated by urate. It has been suggested that elevated urate levels may be sensed by bacterial pathogens, enabling recognition of host environments and eliciting expression of virulence factors (Gupta et al., 2017). Mammalian cells often use xanthine oxidase to generate reactive oxygen species (ROS) to combat bacterial infection, producing urate as a byproduct at levels that can exceed 200 μM (Segal et al., 2000; Martin et al., 2004; Crane et al., 2013). Urate is thought to act by binding to MftR, a transcriptional repressor that negatively regulates ScmR and serves as its partner in regulating secondary metabolite biosynthesis (Gupta et al., 2017). Although MftR itself was not detected in our proteomics data, 32 of the 125 proteins differentially expressed in our study (~26%) are encoded by genes repressed by MftR; these include ScmR and 18/19 genes under its control (Figure 7D, Supplementary Table 2). However, despite the clear correlation between proteins differentially expressed in host-associated bacteria and regulation of their cognate genes by ScmR and urate, we found no such correlation between proteins differentially expressed in host-associated bacteria and regulation of their cognate genes by MftR, indicating that the impact of MftR on protein expression during bacterial infection is more complex than expected.

Using BLASTp (Chen et al., 2016) and the virulence factor database (VFDB), we discovered that 36 of the 125 proteins differentially expressed in host-associated bacteria during infection show significant sequence similarity to previously described bacterial virulence factors (Supplementary Table 2). We found that two of the *Burkholderia* proteins overexpressed during infection (Q2SWC1/PhbB and Q2T838, encoded by BTH_I2257/*phbB* and BTH_II0461, respectively) show strong sequence similarity to CylG, a 3-ketoacyl-ACP reductase that contributes to synthesis of granadaene (an ornithine rhamnolipid pigment with beta-hemolytic and cytolytic activities) as well as to virulence of Group B Streptococcus (GBS) bacteria (Forquin et al., 2007; Whidbey et al., 2013). Q2T4X6 and Q2T4A4 (encoded by BTH_II1578 and BTH_II1801) are likewise overexpressed during *B. thailandensis* infection, but showed sequence similarity to extracellular virulence factors (*Clostridium perfringens* collagenase [kappa-toxin], and *Listeria monocytogenes* adhesion protein, respectively). Q2STQ3 (encoded by BTH_I3202) showed sequence similarity to phospholipase C (PlcH), a hemolytic exotoxin that is secreted by *Pseudomonas aeruginosa* upon interaction with a eukaryotic host (Cota-Gomez et al., 1997). PlcH degrades phospholipids found in cell membranes and lung surfactants, which releases fatty acids and choline-containing compounds that can be repurposed for catabolism by the pathogen (Jackson et al., 2013). Finally, we found that Q2T3C0, Q2T3C1, and Q2T3C2 (encoded by BTH_II2141, BTH_II2140, and BTH_II2139, respectively), which were previously annotated as potentially involved in heme transport, show sequence similarity to the components of two well-characterized heme transport systems: ChuAST of *E. coli* and ShuAST of *Shigella* (Mills and Payne, 1995; Torres and Payne, 1997; Wyckoff et al., 1998). These results suggest that a variety of virulence factors may be differentially expressed during *B. thailandensis* infection.

## Discussion

The intracellular lifestyle of *Burkholderia* spp. plays a central role in enabling bacterial survival within the host and evasion of its immune system. Protein expression profiling of bacteria during host infection is likely to elucidate key molecular mechanisms of intracellular survival; however, proteomic studies of infected host cells are hindered by the overwhelming abundance of host proteins, as compared to bacterial proteins, in infected samples. In concept, selective labeling and enrichment of proteins produced by bacteria during host infection should enable focused analysis of the bacterial proteome to the exclusion of host proteins. The recent advent of bio-orthogonal noncanonical amino acid tagging (BONCAT) in bacteria has provided a powerful tool to study intracellular pathogens by enabling selective enrichment of the bacterial proteome produced within infected host cells.

In this study, we applied the BONCAT method to *B*. *thailandensis* by engineering a strain that expresses a methionyl-tRNA synthetase variant (MetRS^NLL^) that incorporates azidonorleucine (Anl) rather than methionine during protein synthesis. We demonstrated that BONCAT can be used to selectively label the proteome of *B. thailandensis* infecting host cells, and to effectively isolate bacterial proteins from infected cultures. Mass spectrometry analysis of the isolated bacterial proteins allowed for quantitative analysis of the proteome produced by *B. thailandensis*. Additionally, we have shown that Anl-labeled bacterial proteins can be fluorescently tagged for visualization within infected host cells. We used BONCAT to characterize and compare the proteomes of *B. thailandensis* in monoculture *vs*. during host cell infection. Our data provide new insights into the molecular mechanisms underlying the intracellular lifestyle of *Burkholderia* spp.

This first survey of the proteome produced by *B. thailandensis* during infection both addressed and raised complex questions regarding the intracellular lifestyle. For instance, it is not clear from our bacterial proteomics data which host metabolites the bacteria are taking advantage of as carbon and energy sources within the host cell. The overexpressed proteins centered around acetyl-CoA metabolism suggest that the bacteria might be utilizing host lipids. On the other hand, overexpression of glyceraldehyde-3-phosphate dehydrogenase and phosphoenolpyruvate synthetase suggests use of a carbon source that directly feeds into glycolysis instead. Moreover, our observation that *B. thailandensis* reduces expression of several siderophore and heme uptake mechanisms during infection seems to contradict the conventional understanding of the need for intracellular bacteria to compete with the host cell for iron (Andrews et al., 2003; Schaible and Kaufmann, 2004; Leon-Sicairos et al., 2015); on the other hand, it may reflect the bacteria switching to different iron uptake mechanisms due to a difference in iron availability between the host cytosol and the bacterial monoculture condition used for comparison.

Four of the 7 proteins most strongly overexpressed by host-associated *B. thailandensis* are annotated as “Uncharacterized protein” or “Membrane protein, putative” in UniProt, whereas only one out of the 92 proteins underexpressed were comparably annotated. While it is beyond the scope of this study to reannotate all proteins differentially expressed during *B. thailandensis* infection, we reviewed the annotation of the proteins showing the largest changes in expression during infection, which provided some intriguing insights. For example, the protein showing the highest expression level in host-associated bacteria (Q2T6D1, encoded by BTH_II1071) has been annotated as belonging to a “Domain of Unknown Function” protein family DUF849 generally associated with beta-keto acid cleavage enzymes (Bastard et al., 2014); however, recent structural and functional studies have concluded that the protein is Obc1, a bifunctional enzyme that catalyzes quorum sensing-dependent oxalogenesis, which is indispensable for *Burkholderia* survival in stationary phase (Oh et al., 2016). Similarly, the protein showing the highest fold change in overexpression in host-associated bacteria (Q2T3Y0, encoded by BTH_II1925) has been annotated as a “chitin binding domain protein” belonging to a family of lytic polysaccharide monooxygenases that mediate biomass degradation; but in recent years, several members of this family have been shown to contribute to virulence in a variety of bacterial pathogens, including *B. mallei* (A0A0H2WBG2, encoded by BMAA1785) (Frederiksen et al., 2013). Since mammalian cells do not produce chitin, these proteins are thought to act instead on host glycoproteins, glycolipids, or polysaccharides; for instance, they have been shown to mediate mucin binding in *V. cholerae* (Wong et al., 2012), and bacterial adhesion to host epithelial cells (Kawada et al., 2008). Indeed, there is evidence to suggest that chitinases enable the pathogen to suppress host innate immunity (Chaudhuri et al., 2013), consistent with the observation that many proteins of the human immune system are glycosylated (Marth and Grewal, 2008).

**Figure 8:**
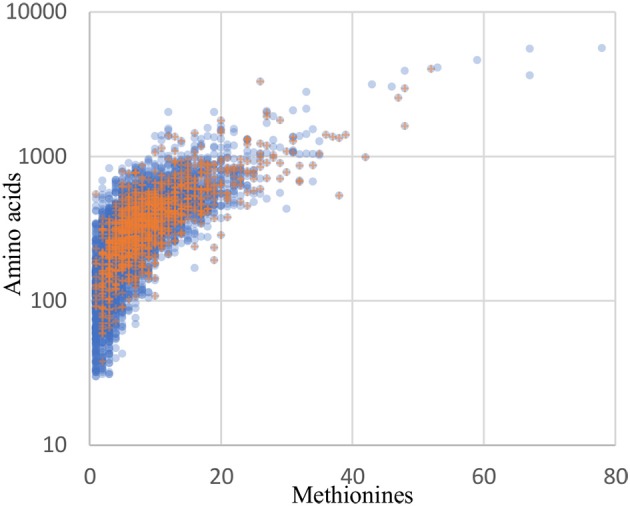
The distribution of recovered proteins reflect the genome distribution well. Blue circles: all 5561 coding sequences from the *B. thailandensis* E264 genome. Orange plus signs: the 1171 proteins detected in the culture and infection biological replicates with at least 2 peptides in any one sample.

Although orthogonal labeling of newly synthesized bacterial proteins proved effective in enabling enrichment of the *B. thailandensis* proteome from infection cultures, the coverage breadth and depth, as well as specific proteins detected, may have been limited by technical aspects of BONCAT. Detection of a protein by this method is not only dependent on the length of the protein but also on the number of methionine residues in its sequence. Protein recovery may therefore be biased toward longer, methionine-rich proteins. Across the entire *B. thailandensis* E264 genome, the median number of methionines *per* protein coding sequence is 6, and only 5% of proteins have a single methionine. This means that we should expect the majority of the proteins to undergo labeling even if *per*-methinonine labeling efficiency is low. The 1171 proteins that are detected in monoculture or during infection do show a small bias in terms of protein sequence length (median of 352 residues, *vs*. 300 for all genome-encoded proteins) and methionine content (median of 8 methionine residues, *vs*. 6 for all proteins). However, aside from that small bias, the overall distribution of detected proteins matches the genome-encoded distribution reasonably well (Figure 8). In addition, our data show that proteome coverage was reduced in samples derived from infected host cells, with approximately half the number of proteins detected relative to the number detected in monoculture. It is therefore difficult to distinguish whether some proteins were undetected due to downregulation or, alternatively, as a result of lower detection sensitivity in the infection condition. In light of these issues, it seems likely that our list of proteins overexpressed by host-associated bacteria is not exhaustive.

Given the successful application of BONCAT to analysis of the proteome produced by *B. thailandensis* during infection, it seems reasonable to expect that this method can be successfully applied to study other aspects of *Burkholderia* pathogenesis. For example, BONCAT could be employed to identify and visualize proteins that are secreted into host cells during infection (e.g., during invasion, escape from the endosome, and/or spread into neighboring host cells *via* cell-cell fusion). A similar approach was taken previously in studies focusing on *Yersinia enterocolitica* and *Mycobacterium tuberculosis* pathogenesis (Mahdavi et al., 2014; Chande et al., 2015). BONCAT should also support proteome profiling of bacteria exposed to different host cell environments (e.g., phagocytic *vs*. non-phagocytic cells), which should further inform our understanding of the molecular mechanisms underlying *Burkholderia* survival within host cells. Amoebae represent yet another niche that could be of relevance for spread and survival of *Burkholderia* bacteria in natural environments (Inglis et al., 2000; DiSalvo et al., 2015). It is even conceivable that the BONCAT method could be successfully used for analysis of *Burkholderia* protein expression *in vivo*, although to our knowledge this has not been reported yet in other infection models.

In summary, we have used the BONCAT method to gain new insight into the proteome produced by *B. thailandensis* during host infection, thereby further informing our understanding of the intracellular lifestyle of *Burkholderia* spp. This study, and future efforts employing this method, will make valuable contributions to our knowledge of the molecular mechanisms underlying *Burkholderia* pathogenesis.

## Author contributions

MF designed and generated reporter strain, generated lab results presented in this paper, helped in analysis of proteomic data, prepared manuscript, corresponding author. ML and YH assisted with transforming of reporter strain. PD contributed with bioinformatic analysis of the data and manuscript preparation. SE-E, BS, and SB participated by giving critical input during the design and troubleshooting of this study, contributed to manuscript preparation.

### Conflict of interest statement

The authors declare that the research was conducted in the absence of any commercial or financial relationships that could be construed as a potential conflict of interest.

## Supplementary material

Supplementary Figure 1 STRING Interaction network between the 125 proteins differentially expressed during infection *vs*. monoculture. *B. thailandensis* proteins were mapped to the *B. pseudomallei* proteins in the STRING database. Colored edges indicate different types of protein-protein associations. Cyan: known interactions from curated databases. Pink: experimentally determined. Green: predicted based on gene neighborhood. Red: predicted based on gene fusions. Blue: predicted based on gene co-occurrence. Olive: co-mentioned in Pubmed abstracts. Black: co-expressed. Purple: protein homology. **(A)** The association network for the 33 proteins overexpressed during infection shows one well-connected component consisting of enzymes involved in butanoate metabolism (red), and valine, leucine and isoleucine degradation (blue). **(B)** The association network of the 92 proteins downregulated in infection condition is densely connected. Red: proteins involved in translation. Green: biosynthesis of amino acids. Blue: biosynthesis of secondary metabolites. The connected component at the bottom right corresponds to a cluster of three siderophore and heme transport systems.

Supplementary Figure 2 Detection of residue-specific incorporation of Anl in Q2T838 (BTH_II0461, Acetoacetyl-CoA reductase). **(A)** Highlighted sequences depict coverage by LC-MS/MS; modified Met residues are highlighted in green indicating incorporation of Anl. **(B)** Representative peptide spectrum from an LC-MS/MS analysis on a precursor ion of the TDLDAM+23FNVTK peptide, outlined in a box in A. The mass shift associated with replacement of Methionine with Azidonorleucine corresponds to 23 amu, depicted as “M+23” in the spectrum (b ions in red, y ions in blue). **(C)** Representative spectrum of the peptide without the Met->Anl substitution.

Supplementary Table 1 List of all identified proteins under monoculture and infection conditions. The list of all identified proteins and their spectral counts in three biological replicates of monoculture and infection conditions.

Supplementary Table 2 Complete list of all differentially expressed proteins in infection *vs*. monoculture conditions. Columns from left to right: Gene: Uniprot accession number; Locus: Locus tag; Identified proteins: Uniprot protein name; STRING annotation: Annotation of homologous *B. pseudomallei* protein in STRING; Log2fold: Log-2 fold protein overexpression infection *vs*. monoculture, based on DESeq2 analysis; padj: FDR-adjusted p-value of differential expression infection *vs*. monoculture, based on DESeq2 analysis; Monoculture 1-3: DESeq2 normalized spectral count for monoculture biological replicate 1-3; Infection 1-3: DESeq2 normalized spectral count for infection biological replicate 1-3; ΔscmR: Log-2 fold change in gene expression in scmR deletion mutant *vs*. WT, based on (Mao et al., 2017); ΔmftR: Log-2 fold change in gene expression in mftR deletion mutant *vs*. WT, based on (Gupta et al., 2017); Urate: Log-2 fold change in gene expression in WT cells grown with 5 mM urate, based on (Gupta et al., 2017); Phase: Gene expression Quorum-Sensing regulated at the transition from logarithmic growth to stationary phase (T phase) or stationary phase (S phase), based on (Majerczyk et al., 2014); WT: Log-2 fold-change in gene expression in the wild-type strain compared to AHL- mutant JRC112 without added AHLs, based on (Majerczyk et al., 2014); QS-1-3: Log-2 fold-change in gene expression when adding the AHL for QS-1-3 to AHL- mutant JRC112, based on (Majerczyk et al., 2014); All: Log-2 fold-change in gene expression when adding the AHLs for all three QS systems to AHL- mutant JRC112, based on (Majerczyk et al., 2014); VFDB BLASTP: Closest BLASTp hit to known virulence factors in the core dataset of the Virulence Factors Database (VFDB, http://www.mgc.ac.cn/VFs/).
