# Supplementary figures and images for: Proteomic Profiling of Burkholderia thailandensis During Host Infection Using Bio-Orthogonal Noncanonical Amino Acid Tagging (BONCAT)
Source: Front Cell Infect Microbiol. 2018 Oct 23;8:370. doi: 10.3389/fcimb.2018.00370 (PMC6206043; doi:10.3389/fcimb.2018.00370)

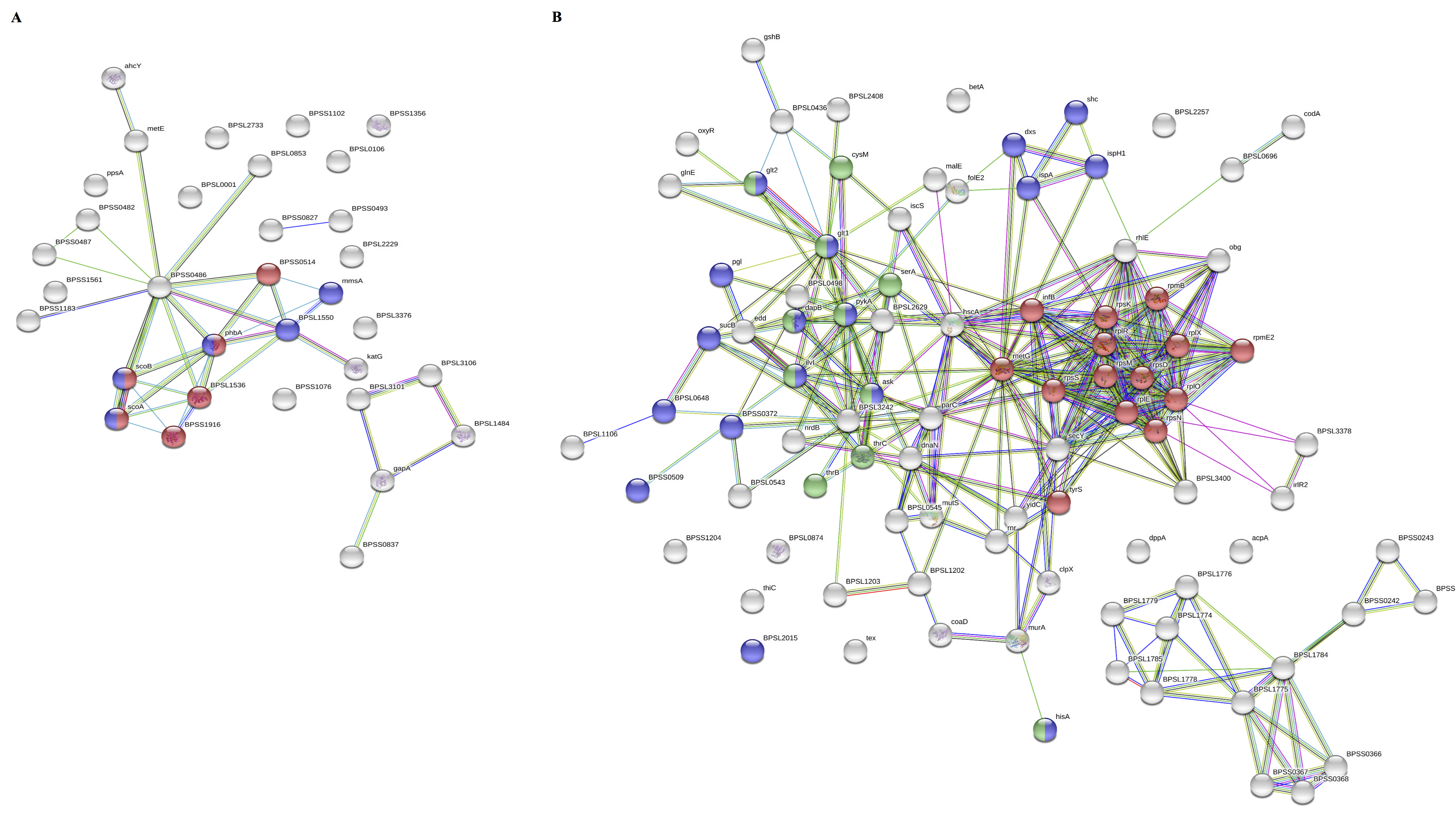

Supplement: Supplementary Figure 1 — STRING Interaction network between the 125 proteins differentially expressed during infection vs. monoculture. B. thailandensis proteins were mapped to the B. pseudomallei proteins in the STRING database. Colored edges indicate different types of protein-protein associations. Cyan: known interactions from curated databases. Pink: experimentally determined. Green: predicted based on gene neighborhood. Red: predicted based on gene fusions. Blue: predicted based on gene co-occurrence. Olive: co-mentioned in Pubmed abstracts. Black: co-expressed. Purple: protein homology. (A) The association network for the 33 proteins overexpressed during infection shows one well-connected component consisting of enzymes involved in butanoate metabolism (red), and valine, leucine and isoleucine degradation (blue). (B) The association network of the 92 proteins downregulated in infection condition is densely connected. Red: proteins involved in translation. Green: biosynthesis of amino acids. Blue: biosynthesis of secondary metabolites. The connected component at the bottom right corresponds to a cluster of three siderophore and heme transport systems. [file Image_1.JPEG]

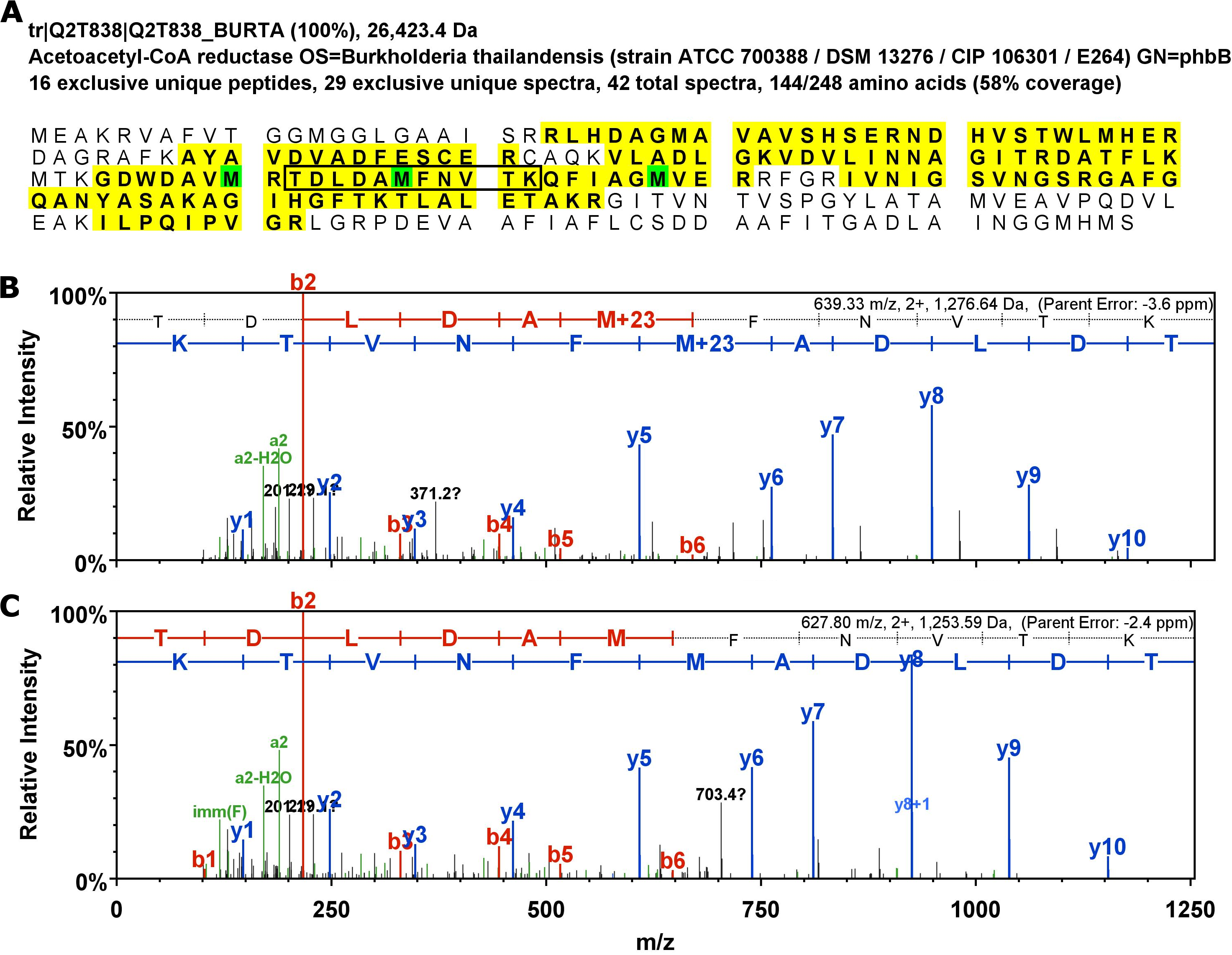

Supplement: Supplementary Figure 2 — Detection of residue-specific incorporation of Anl in Q2T838 (BTH_II0461, Acetoacetyl-CoA reductase). (A) Highlighted sequences depict coverage by LC-MS/MS; modified Met residues are highlighted in green indicating incorporation of Anl. (B) Representative peptide spectrum from an LC-MS/MS analysis on a precursor ion of the TDLDAM+23FNVTK peptide, outlined in a box in A. The mass shift associated with replacement of Methionine with Azidonorleucine corresponds to 23 amu, depicted as “M+23” in the spectrum (b ions in red, y ions in blue). (C) Representative spectrum of the peptide without the Met->Anl substitution. [file Image_2.TIFF]
